# Alternative Splicing Mechanisms Underlying Opioid-Induced Hyperalgesia

**DOI:** 10.3390/genes12101570

**Published:** 2021-10-01

**Authors:** Pan Zhang, Olivia C. Perez, Bruce R. Southey, Jonathan V. Sweedler, Amynah A. Pradhan, Sandra L. Rodriguez-Zas

**Affiliations:** 1 Illinois Informatics Institute, University of Illinois at Urbana-Champaign, Urbana, IL 61801, USA; panz2@illinois.edu; 2 Department of Animal Sciences, University of Illinois at Urbana-Champaign, Urbana, IL 61801, USA; ocperez2@illinois.edu (O.C.P.); southey@illinois.edu (B.R.S.); 3 Department of Chemistry and the Beckman Institute, University of Illinois at Urbana-Champaign, Urbana, IL 61801, USA; jsweedle@illinois.edu; 4 Department of Psychiatry, University of Illinois at Chicago, Chicago, IL 60612, USA; pradhan4@uic.edu; 5 Department of Statistics, University of Illinois at Urbana-Champaign, Urbana, IL 61801, USA

**Keywords:** morphine, transcript isoform, glutamatergic system, transcription factor

## Abstract

Prolonged use of opioids can cause opioid-induced hyperalgesia (OIH). The impact of alternative splicing on OIH remains partially characterized. A study of the absolute and relative modes of action of alternative splicing further the understanding of the molecular mechanisms underlying OIH. Differential absolute and relative isoform profiles were detected in the trigeminal ganglia and nucleus accumbens of mice presenting OIH behaviors elicited by chronic morphine administration relative to control mice. Genes that participate in glutamatergic synapse (e.g., Grip1, Grin1, Wnk3), myelin protein processes (e.g., Mbp, Mpz), and axon guidance presented absolute and relative splicing associated with OIH. Splicing of genes in the gonadotropin-releasing hormone receptor pathway was detected in the nucleus accumbens while splicing in the vascular endothelial growth factor, endogenous cannabinoid signaling, circadian clock system, and metabotropic glutamate receptor pathways was detected in the trigeminal ganglia. A notable finding was the prevalence of alternatively spliced transcription factors and regulators (e.g., Ciart, Ablim2, Pbx1, Arntl2) in the trigeminal ganglia. Insights into the nociceptive and antinociceptive modulatory action of Hnrnpk were gained. The results from our study highlight the impact of alternative splicing and transcriptional regulators on OIH and expose the need for isoform-level research to advance the understanding of morphine-associated hyperalgesia.

## 1. Introduction

Chronic pain affects 11% to 40% of the United States population [1,2]. A common treatment for pain is short-term opioid-based therapies, which can amend pain while diminishing the risk of addiction. However, the prolonged use of opioids can lead to opioid-induced hyperalgesia (OIH), a condition characterized by oversensitivity to a noxious stimuli that could lead to an increase in opioid consumption [3,4]. Akin to neuropathic pain, OIH increases the sensitivity of primary and secondary neurons such as those located in the dorsal root ganglion and dorsal horn pertaining to the afferent pathway [5].

Rodent models of OIH have advanced the understanding of the central nervous system (CNS) regions and molecular mechanisms underlying OIH [6,7,8,9,10,11]. Rodents repeatedly administered morphine presented hypersensitivity to noxious stimuli evidenced by the higher mechanical or thermal sensitivity to von Frey filaments and hot plate tests [7,9], and alterations of molecular pathways [6,10,11,12]. For example, our studies of chronic morphine exposure in mice detected changes the abundance of numerous neuropeptides across multiple CNS regions [6]. We also detected changes in the expression of genes in the circadian rhythm and adaptive immune response pathways in the trigeminal ganglia (TG), a brain region that processes and transmits stimuli signals, and the nucleus accumbens (NAc), a brain region associated with opioid response [10]. Prolonged opioid use changes myelin proteins and genes and proteins that participate in glutamatergic synapse processes [10,13,14]. Chronic opioid exposure increases glutamate availability to ion channel N-methyl-D-aspartate (NMDA) receptors, and activates NMDA receptors and protein kinases that in turn further upregulate NMDA receptors and downregulate opioid receptors. The previous process, known as the “second messenger switching”, enhances the transmission of OIH stimuli signals [13,14].

Alternative splicing can regulate gene expression and action through the production of transcript isoforms that differ in their capability to generate biologically active products [15,16]. Differential abundance of splice variants annotated to receptors, ion channels and growth factors associated with nociception and pain have been reported [17,18,19]. Our targeted study of circadian rhythm and toll-like receptor networks demonstrated the transcript isoform profiling can offer additional insights on OIH [12]. Moreover, alternative splicing impacts the function of genes in many pathways involved in OIH such as glutamatergic and myelination processes. For example, the alternative splicing of Grin1, a gene that encodes a subunit of the NMDA receptor, has been associated with spatial memory acquisition [20]. Alternative splicing also affects the expression of myelin protein components and myelination patterns associated with multiple sclerosis [21]. 

Despite the established effects of alternative splicing on genes annotated to pathways associated with OIH (e.g., glutamatergic, myelination), the impact of alternative splicing in OIH remains partially characterized. The overarching goal of this study is to gain a comprehensive understanding of the effects of alternative splicing on OIH. The supporting objectives are, a) the characterization of alternative splicing action modes associated with OIH that are region-dependent or ubiquitous of the region, and b) to uncover pathways predominantly impacted by alternative splicing. The findings from our study aid in the identification of transcript isoforms associated with OIH, therefore improving the accuracy of detecting effective therapeutic targets to ameliorate OIH.

## 2. Materials and Methods

Animal experimental protocols were approved by the Office of Animal Care and Institutional Biosafety Committee at the University of Illinois at Chicago in accordance with AALAC guidelines, the Animal Care Policies of the University of Illinois at Chicago, and the NIH Public Health Service Policy on the Humane Care and Use of Animals.

A bulk RNA-sequencing experiment was undertaken to measure the level of gene expression in the NAc and TG of male adult C57BL6/J mice administered with morphine to elicit OIH (OIH group) relative to control mice (CON group) [10]. The animal experiments and the results from the standard analysis of expression at the gene level have been previously reported [10]. The 20 samples available from individual mice were equally distributed between treatments (OIH and CON groups) and between brain regions (NAc and TG). Mice did not exposed to conditions that elicit pain prior to the trial and the OIH group were subcutaneously administered morphine at a dose of 20 mg/kg twice a day in days 1-3 and 40 mg/kg on day 4 in recognition of possible tolerance development. The same 0.9% saline vehicle used to dilute the morphine was administered to the control group at the same time as the OIH group. Under the previous protocol of morphine administration, the hypersensitivity to mechanical stimuli from Von Frey filaments in the hind paw and cephalic regions is significantly higher in the OIH relative to the control by the third day of the trial and remains heightened even on day 5 [6,10]. The two CNS regions from each mouse were collected 24 hrs after the final vehicle or morphine injection. In the morning of the fifth day, mice were anesthetized by Somnasol injection, intracardially perfused with ice-cold PBS buffer, decapitated and the TG and NAc were extracted. For each mouse and brain region, tissue samples were homogenized and the RNA was isolated. All samples had a RNA integrity > 7.5, and the libraries were sequenced using a HiSeq 4000 platform (Illumina, San Diego, CA, USA) generating 100 bp-long pair end reads. The sequencing protocol yielded a coverage of ~ 16 million reads per sample corresponding to a mouse and CNS region. The FASTQ sequence files are available in the Gene Expression Omnibus (GEO) database (experiment identifier GSE126662) and were not trimmed because all positions had a Phred quality score > 30 [22,23].

The sequenced reads from each sample were aligned to the C57Bl/6J mouse genome (version GRCm38) [24] using STAR (v.2.5.3a) in two-pass mode to further enhance the accuracy of the individual transcript isoform alignment [25]. The isoform-level output from STAR was subsequently processed in RSEM (v.1.3.1) to quantify the transcript isoform expression in raw reads counts [26]. This pipeline differed from our previous analysis [10,12,23] and optimized the quantification of expression at the transcript isoform level. 

### 2.1. Dual Analysis of Absolute and Relative Alternative Splicing

A comprehensive characterization of the role of alternative splicing on OIH was gained by studying absolute and relative mode of actions. The absolute differential analysis was implemented on a per-transcript isoform basis and enabled us to assess the overall splicing effect that resulted in the change in the expression of individual transcript isoforms between the OIH and control groups for each region. However, the quantification of changes in absolute transcript isoform expression cannot offer information into changes in the transcript isoform proportion between treatments. Therefore, a second analysis was undertaken to test for relative differential isoform expression.

The absolute differential isoform expression between the OIH and control groups within region was tested using the edgeR (v.3.14.0) [27] in the R environment (v.3.3.1) on the trimmed mean of M values normalized transcript isoform counts. Transcript isoforms that had at least 5 reads per treatment-region group were tested for differential expression and the Benjamini-Hochberg false discovery rate (FDR) was used to adjust the test-statistic *P*-value for multiple testing [28]. The criteria to identify significant absolute differential isoform expression encompassed FDR-adjusted *P*-value and minimum log2(fold change between OIH and control groups).

The relative differential isoform expression between the OIH and control groups within region was tested using LeafCutter (v.0.2.9) [29].The clustering of overlapping transcript introns provides an estimate of transcript intron usage, and the Dirichlet-multinomial model implemented in LeafCutter enables the testing for differentially intron excision and thereof transcript isoform [29]. The LeafCutter specifications included, >50 split reads per cluster, intron length <500 kb, and intron clusters detected in >3 samples per treatment-region group. The Percentage Spliced Index (PSI) was used to measure the relative expression of each transcript isoform within a treatment group and region with respect to the total expression across all transcript isoforms in the cluster. The PSI is a frequently used metric to characterize alternative splicing [30,31] and is computed based on the number of RNA-seq reads that support a particular exon or group of exons in a transcript isoform. The change in PSI (ΔPSI) was used to quantify the relative differential expression of each transcript isoform between the OIH and control mice and the statistical test for differential splicing combines the ΔPSI across all transcript isoforms within a cluster to provide one *P*-value per gene intron cluster [11,12]. Multiple isoform clusters may be tested at different locations within a gene and genome locations annotated to multiple gene will share test results. The criteria to identify significant relative differential isoform expression encompassed FDR-adjusted *P*-value and minimum ΔPSI between OIH and control groups. The detection of significant alternative splicing encompassed the simultaneous consideration of the overall *P*-value and minimum ΔPSI of a gene. The series of introns representing transcript isoforms within a cluster and the corresponding mapped genes were visualized using Gencode M18 mouse genome reference annotation [32]. 

The complementary insights gained on OIH stem from the distinct description of alternative splicing offered by the two analyses considered. The absolute analysis models each annotated isoform profile independent from the other isoforms in the gene and allows testing for significant changes between OIH and control. The absolute analysis of annotated isoforms employs known exon usage information, therefore enabling the alignment and benchmarking of the present isoform-level analysis against the previously published gene-level analysis [10]. The relative analysis models each isoform identified through intron clusters that were determined from intron/exon junction information relative to all other detected isoforms. Therefore, the relative analysis required the computation of the total abundance across all detected isoforms to express each isoform as a proportion. Typical of high throughput RNA-seq studies, we detected isoforms both already annotated to genes, and currently unannotated, requiring a minimum of 50 split reads mapped to each cluster and maximum intron length of 500 kb. The relative analysis enabled the detection of changes in proportional isoform representation, irrespective of previous annotation.

### 2.2. Functional Enrichment

The impact of OIH on the absolute and relative alternative splicing of genes within PANTHER pathways [33] in the NAc and TG were studied using WebGESTALT v.2019 (http://www.webgestalt.org/, accessed on 1 May 2021) enrichment routines [34]. Two complementary approaches were used to identify the enrichment of functional categories, the Over-Representation Analysis (ORA) and Gene Set Enrichment Analysis (GSEA). The ORA analysis uses the hypergeometric test to assess the enrichment of a functional category [35,36], and differentially absolute or relative transcript isoform expression between OIH and control groups at *P*-value < 0.0005, irrespectively of the profile sign (over- or under-expression in OIH relative to control) were considered in the analysis. The ORA approach was also used to identify enrichment of Gene Ontology Biological Processes [37] in the list of genes that presented both splicing modes of action within a region or that presented the same splicing mode of action across two regions.

The GSEA considered the profile of all transcript isoforms tested, and the input score was the signed –log10(*P*-value) of absolute or relative differential expression. The GSEA of absolute differential expression between OIH and control uses the sign of each transcript isoform, where a positive log2(fold change) indicates over-expression, and a negative log2(fold change) indicates under-expression in OIH relative to control mice.

A strategy was developed to study the pathway enrichment in consideration of the simultaneous over- and under-expression of isoforms within a gene in the relative alternative splicing approach. The GSEA of the relative expression (proportions) of the transcript isoforms in a cluster encompasses both signs because some transcript isoforms are over-abundant relative to the remaining isoforms that are under-abundant in OIH versus control mice. The sign assigned to the GSEA input score for each transcript isoform cluster was the sign of the most extreme ΔPSI between OIH and control. Therefore, a positive GSEA score sign indicates that the transcript isoform presenting the most extreme change was over-expressed in OIH relative to control mice, while a negative GSEA sign indicates that the most extreme transcript isoform in the cluster was under-expressed in OIH relative to control mice.

The enrichment tests of the absolute and relative analysis and the background genome were implemented at the gene level with the goal of identifying pathways with prevalent alternative splicing processes. Both ORA and GSEA provide a *P*-value and FDR-adjusted *P*-value of the pathway enrichment. ORA uses hypergeometric test and computes an enrichment ratio (observed versus expected). GSEA computes the normalized enrichment score (NES) based on the maximum deviation of the cumulative sum based on the signed fold-change divided by the average of the permutated enrichment scores (based on 2000 permutations). *Mus musculus* was used as the reference genome, and the enrichment analysis limited the pathways with a minimum of 5 detected genes and a maximum of 2000 genes per category. 

### 2.3. Gene and Transcription Factor Network Reconstruction

To understand the interaction between the genes presenting absolute or relative alternative splicing mode of action associated with OIH within region, we reconstructed networks depicting known relationships between the genes using the BisoGenet plug-in [38] within Cytoscape (v.3.8.2) [39]. The network nodes correspond to genes and the edges correspond to protein-protein interactions obtained by BisoGenet from the BIOGRID, DIP, BIND, HPRD, DIP, BIND, INTACT, and MINT databases [40,41,42,43,44,45]. The network framework includes genes that presented absolute or relative differential splicing (*P*-value < 0.005, |fold change| > 2) between OIH and control mice. To facilitate the visualization of the networks, only direct interactions (edges) connecting two genes (nodes) that have significant differential isoform expression or splicing are depicted. While the reconstructed network enables the detection of molecular relationship between alternative spliced or expressed, the hub genes presenting high connectivity with other genes that may play an important role in the development or maintenance of OIH. 

In addition to studying the relationship between alternatively spliced genes associated with OIH based on protein-protein interactions, the relationships between these genes through common transcription factor were also investigated. Genes that presented absolute or relative differential splicing between OIH and control (*P*-value < 0.005, |fold change| > 2) were queried for enriched transcription factors (TFs) using the *iRegulon* [46] plugin within Cytoscape. The genes presenting differential alternative splicing were searched against the database of target genes ranked according to the similarity to the corresponding transcription factors binding motifs. Transcription factor enrichment scores were computed based on the area under the cumulative recovery curve obtained from the database search.

## 3. Results

### 3.1. Absolute Differential Expression of Isoforms Associated with Opioid-Induced Hyperalgesia

Overall, 263 transcript isoforms from 250 genes and 363 transcript isoforms from 319 genes present absolute differential expression (FDR-adjusted *P*-value < 0.05) between OIH and control mice in the NAc and TG, respectively. The absolute differentially gene expression between OIH and control mice at FDR-adjusted *P*-value < 5.0 × 10^–5^ and |fold change| > 2 in NAc and TG are presented in Table 1 and Table 2, respectively. The extended lists of transcript isoforms presenting absolute differential expression associated with OIH at *P*-value < 0.05 in NAc and TG are provided in Supplementary Tables S1 and S2, respectively. 

### 3.2. Relative Differential Expression of Isoform Associated with Opioid-Induced Hyperalgesia

Overall, 9 genes presented significant (FDR-adjusted *P*-value < 0.05) relative differential expression of isoforms between OIH and control mice. Table 3 lists the isoform clusters within genes presenting relative differential expression (FDR-adjusted *P*-value < 0.1) between OIH and control mice including the most extreme positive and negative ΔPSI between the two groups in the NAc and TG. A positive ΔPSI identifies a transcript isoform present in higher proportion in OIH relative to control mice, while a negative ΔPSI identifies a transcript isoform present in lower proportion in OIH relative to control mice. Relative differential splicing was detected at *P*-value < 0.0005 in 40 genes in the NAc and 32 genes in the TG (Supplementary Table S3). The TG presented more extreme splicing (characterized by higher differences in the transcript isoform proportions between OIH and control) relative to NAc (7 versus 2 genes at FDR-adjusted *P*-value < 0.05, Table 3), whereas NAc presented a higher number of relative differential splicing genes at *P*-value < 0.0005 than TG (40 versus 32 genes, respectively, Supplementary Table S3). 

**Figure 1:**
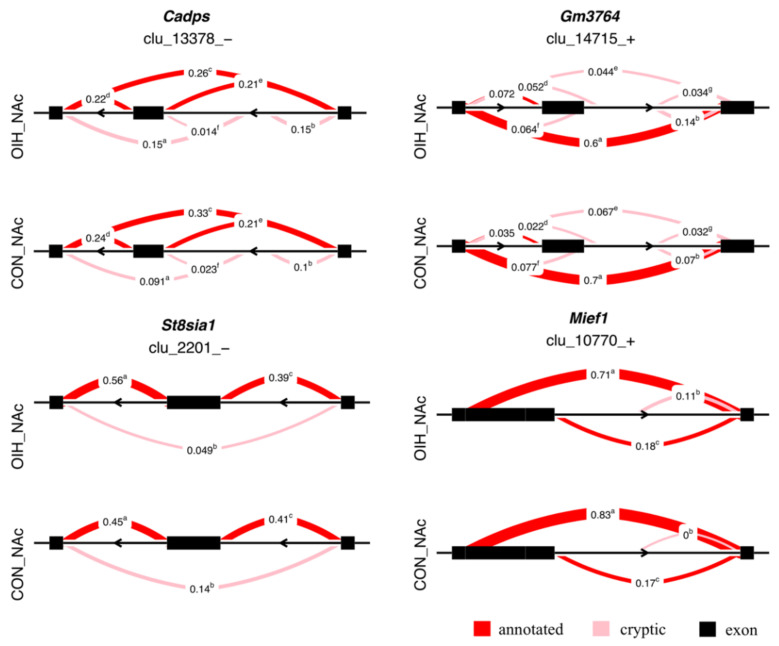
Examples of differential spliced genes (maximum |ΔPSI| > 0.1, *P*-value < 0.005) between opioid-induced hyperalgesia (OIH) and control (CON) mice in the nucleus accumbens (NAc). Genes included Cadp =calcium dependent secretion activator, GM3764=long intergenic non-coding RNA; St8sia1=ST8 alpha-N-acetyl-neuraminide alpha-2,8-sialyltransferase 1; Mief1=mitochondrial elongation factor 1. The semicircles indicate the clusters of introns corresponding to transcript isoforms, the numbers represent the relative proportion of each intron cluster within OIH and control, and the superscript denotes different clusters.

Figure 1 depicts the proportional isoform expression within four gene clusters, calcium-dependent secretion activator 1 (Cadps), a long intergenic noncoding RNA (GM3764), sialyltransferase 8 (St8sia1), and mitochondrial dynamics protein MID51 (Mief1), that presented significant relative differential expression (|ΔPSI| ≥ 0.1, FDR-adjusted P-value < 0.1) in the NAc. The significant differential splicing identified in the NAc for Cadps, was characterized by the under-expression of an isoform that excludes an intermediate exon in OIH relative to control mice with the proportion ratio of 0.26 (OIH):0.33 (control). The significant differential splicing identified in the NAc for St8sia1, was characterized by the over-expression of an isoform that solely includes a 3′ exon in OIH relative to control mice with the proportion ratio of 0.56 (OIH):0.45 (control). The significant differential splicing identified in the NAc for Mief1, was characterized by the under-expression in OIH relative to control mice of an isoform that includes a lengthy 5′ exon with an proportion ratio of 0.71 (OIH):0.83 (control).

**Figure 2:**
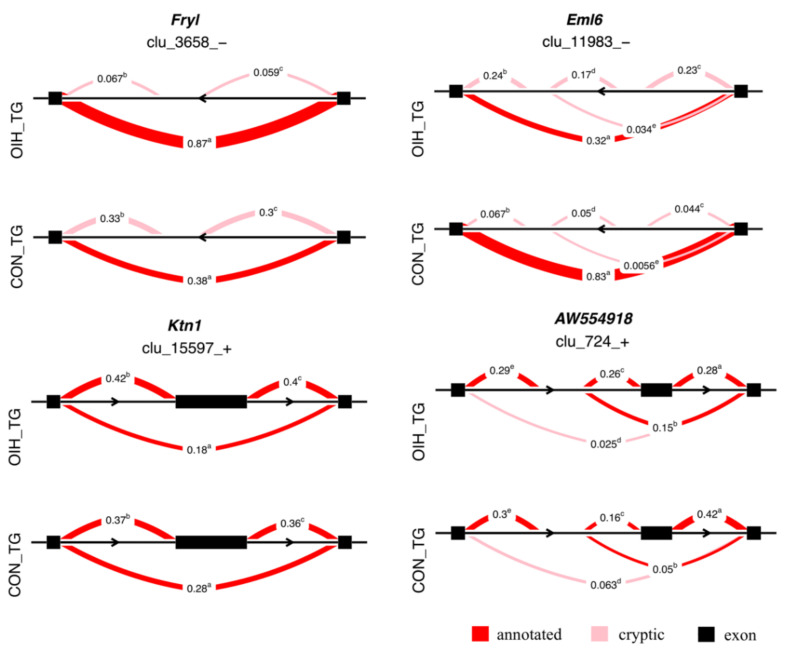
Examples of differential spliced genes (maximum |ΔPSI| > 0.1, *P*-value < 0.005) between opioid-induced hyperalgesia (OIH) and control (CON) mice in the trigeminal ganglia (TG). Genes included Fryl = FRY like transcription coactivator, Eml6 = EMAP like 6, Ktn1=kinectin 1, and expressed sequence AW554918. The semicircles indicate the clusters of introns corresponding to transcript isoforms, the numbers represent the relative proportion of each intron cluster within OIH and control, and the superscript denotes different clusters.

Figure 2 depicts the proportional isoform expression of four genes, FRY-like transcription coactivator (Fryl), echinoderm microtubule-associated protein-like 6 (Eml6), kinectin (Ktn1), and expressed sequence AW554918, that presented significant relative differential expression (|ΔPSI| ≥ 0.1, FDR-adjusted *P*-value < 0.1) in the TG. The relative differential splicing identified in the TG for Fryl, was characterized by the over-expression of an isoform that extends across two exons in OIH relative to control mice with the exon-skipping isoform presenting a proportion ratio of 0.87(OIH):0.36(control). The significant differential splicing identified in the TG for Eml6 was characterized by the under-expression of an isoform that extends across two exons in OIH relative to control mice with a proportion ratio 0.32(OIH):0.83(control). The significant relative differential splicing identified in the TG for Ktn1, was characterized by the under-expression of an isoform that extends across two exons in OIH relative to control mice with a proportion ratio 0.18(OIH):0.28(control). The significant relative differential splicing identified in the TG for AW554918, was characterized by the under-expression of an isoform that extends across two 3′ exons in OIH relative to control mice with a proportion ratio 0.28 (OIH):0.42 (control).

### 3.3. Functional Analysis of Absolute and Relative Differential Isoform Expression Associated with Opioid-Induced Hyperalgesia

Enriched PANTHER pathways (*P*-value < 0.001) associated with the effects of OIH on absolute and relative differential isoform expression between OIH and control mice in the NAc and TG identified using the complementary ORA and GSEA analyses are listed in Table 4. An extended list of enriched pathways within region and type of differential alternative splicing (absolute and relative) is available in Supplementary Table S4. Pathways that presented alternative splicing in both regions and pathways that presented alternative splicing in one region were detected. Among the pathways that present alternative splicing associated with OIH in both regions were platelet derived growth factor, p38 MAPK, axon guidance, and integrin signaling. The gonadotropin-releasing hormone receptor pathway was uniquely enriched in the NAc, whereas vascular endothelial growth factor and endogenous cannabinoid signaling, circadian clock system, and metabotropic glutamate receptor group I pathways were uniquely enriched in the TG.

### 3.4. Integrated Analysis of Absolute and Relative Differential Isoform Expression Associated with Opioid-Induced Hyperalgesia

**Figure 3:**
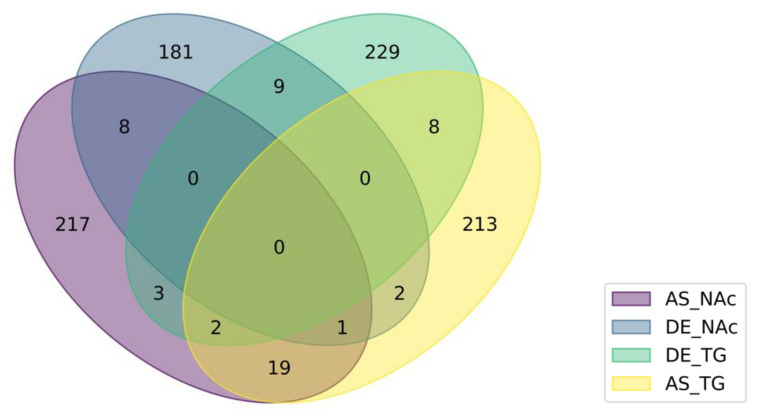
Number of genes presenting absolute (DE) or relative (AS) differential splicing action mode between opioid-induced hyperalgesia and control mice in the nucleus accumbens (NAc) or trigeminal ganglia (TG).

The study of simultaneous changes in absolute and relative transcript isoform expression enabled us to understand the predominant alternative splicing mode of action. Figure 3 depicts the number of genes in each brain region presenting either alternative splicing mode (i.e., absolute or relative differential expression) associated with OIH (*P*-value < 0.005). Table 5 enumerates the genes that correspond to the intersection of ovals in Figure 3 and Table 6 list the enriched Gene Ontology biological processes among the genes in Table 5. The genes in Table 5 and Table 6 presented two splicing action modes within a region or one action mode across two regions. 

The majority of genes had one alternative splicing mode of action (i.e., absolute or relative expression change) and the splicing was expressed in one region (Figure 3). Nine out of 442 genes (2.04%) in the NAc and 10 out of 486 genes (2.06%) in the TG presented both absolute and relative isoform changes between OIH and control mice (Figure 3 and Table 5). Two enriched categories were identified by ORA among the genes that had simultaneous absolute and relative differential expression in NAc (Table 6), including RNA processing (GO:0006396), gene expression (GO:0010467) and positive regulation of nitrogen compound metabolic process (GO:0051173). In addition, 9 genes exhibited OIH treatment effect in NAc and TG, while 22 genes alternative spliced both in NAc and TG.

The enrichment of mRNA metabolic and RNA splicing processes were detected among the genes that presented two splicing mode of actions in one region or one mode of action across two regions (Table 6). The enrichment of the mRNA metabolic process (GO:0016071) encompassed nine genes (Cnot1, Csde1, Eef1d, Celf3, Prpf40b, Rbm3, Rbm39, Hnrnpk, Hnrnpa1), while the enrichment of the process RNA splicing (GO:0008380) was supported by six genes (Celf3, Prpf40b, Rbm3, Rbm39, Hnrnpk, Hnrnpa1). The enrichment of the process regulation of neurogenesis (GO:0050767) included 11 genes (Asap1, Cdon, Clasp2, Grin1, Hnrnpk, Map2, Mapk8ip3, Pak3, Sema4c, Spag9, and Tenm4) and the enrichment of the process positive regulation of the MAPK cascade (GO:0043410) was supported by six genes (Cdon, Mapk8ip3, Mink1, Pak3, Plce1, Sema4c, and Spag9).

### 3.5. Interactions between Genes That Present Absolute and Relative Differential Isoform Expression Associated with Opioid-Induced Hyperalgesia

**Figure 4:**
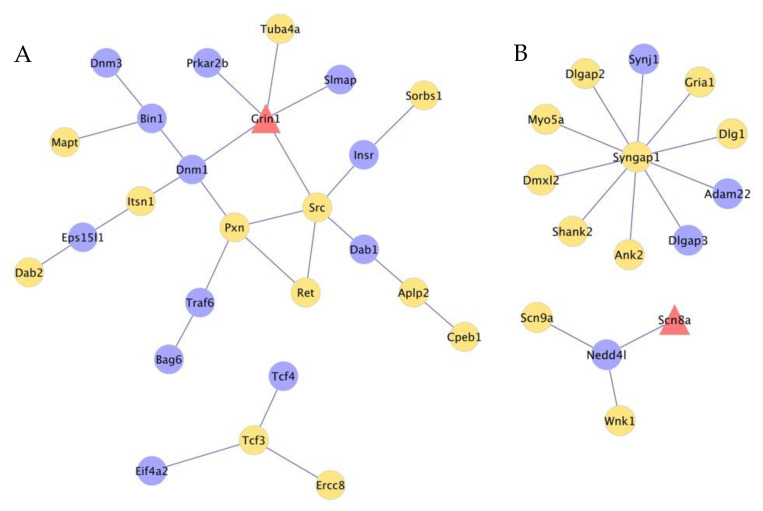
Network of genes presenting absolute or relative differential splicing between opioid-induced hyperalgesia and control mice in the nucleus accumbens (**A**) and trigeminal ganglia (**B**) Edges denote known gene relationships, and node color denote: red = genes presenting absolute and relative differential splicing; yellow = genes presenting absolute differential splicing; and blue = genes presenting relative differential splicing. Gene symbols: In the NAc Tuba4a, Prkar2b, Grin1, Slmap, Scrc, Insr, Sorbs1, Dab1, Aplp2, Cpeb1, Ret, Pxn, Prkar2b, Pxn, Dnm1, Bin1, Dnm3, Mapt, Itsn1, Eps14l1, Dab2, Traf6, Bag6, Tcf3, Tcf4, Eif4a2, Ercc8; In the TG Synj1, Gria1, Dig1, Adam22, Dlgap3, Ank2, Shank2, Dmxl2, Myo5a, Dlgap2, Nedd4al, Scn8a, Scn9a, Wnk1.

The connectivity among genes that presented absolute or relative differential splicing was higher in NAc than in TG (Figure 4). In the NAc network, four genes have high connectivity (nodes connected to (≥4 nodes) and include glutamate receptor ionotropic, NMDA 1 (Grin1), neuronal proto-oncogene tyrosine-protein kinase Src (Src), dynamin-1 (Dnm1), and paxillin (Pxn). Among these genes, Grin1 presented both absolute and relative differential splicing in NAc (Figure 4A).

Highly connected genes in the TG network include Ras/Rap GTPase-activating protein SynGAP (Syngap1), E3 ubiquitin-protein ligase NEDD4-like (Nedd4l), and sodium channel protein type 8 subunit alpha (Scn8a) that presented both absolute and relative differential splicing (Figure 4B). Other genes in the TG network are annotated to transcription regulation, including transcription factor 4 (Tcf4), transcription factor E2-alpha (Tcf3), DNA excision repair protein ERCC-8 (Ercc8), and eukaryotic initiation factor 4A-II (Eif4a2). Table 3. *P*-values of the effects of gestational immune activation (PRRSV-challenged or control gilt) by weaning group (nursed or weaned), and sex on the chemistry analyte and cortisol concentrations and body weights of 22-day-old pigs.

**Figure 5:**
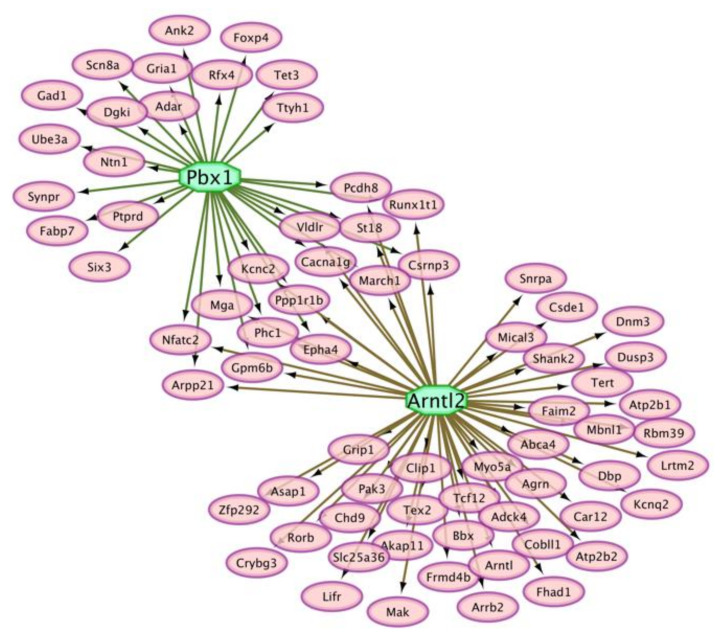
Enriched transcription factors (octagons) including motifs on the regulatory regions of genes (ovals) that presented absolute or relative differential splicing between opioid-induced hyperalgesia and control mice in the trigeminal ganglia.

The TG network (Figure 4B) included relationships among differentially spliced transcription factors and target genes associated with OIH. Motivated by the extensive interaction between transcription factors and alternatively spliced genes associated with OIH in the TG, the over-representation of potential transcription factors among target genes that presented differential splicing was investigated. Table 7 lists additional transcriptional regulators that presented differential splicing in the TG (i.e., Cnot, Rbm39, Ablim2, Cdon, Ciart, Eef1d, Grn1, Hnmpk, Peg3, Ssbp2, and Zfp948). Transcription factor pre-B-cell leukemia transcription factor 1 (Pbx1) and Aryl hydrocarbon receptor nuclear translocator-like protein 2 (Arntl2) were enriched among target genes presenting absolute and relative differential isoform expression between OIH and control mice in the TG. Figure 5 depicts the enriched transcription factors and the target genes that presented absolute or relative differential splicing (*P*-value <0.005 and |fold change between treatments| >1).

## 4. Discussion

Although morphine is typically prescribed for the analgesic effect, repeated morphine administration can trigger the transition from analgesia to hyperalgesia and hypersensitivity to stimuli (i.e., OIH). Repeated administration of morphine elicits OIH in mice, characterized by lower mechanical stimuli threshold [6,10]. Studies by other groups and by us have established relationships between gene expression levels and OIH. The present study pioneers the understanding of alternative splicing action modes associated with OIH across the transcriptome. A strategy that combined the study of absolute differential expression of individual transcript isoforms and relative differential expression of isoforms within a gene enabled us to gain additional understanding of the changes in alternative splicing action modes underlying OIH.

Altogether, the results of the absolute and relative analysis in Table 1 to Table 3 highlight approximately 75 genes presenting changes in the isoform profiles associated with OIH. The overlap between the genes identified by the absolute and relative analysis was limited and eight loci (Tusc5, Pirt, Sncg, Mpz, Acpp, Dbp, Ciart, Arntl) that exhibited differential expression at the gene level [10]. This finding points to the complementary nature of the descriptors of transcript levels considered in this study. The absolute analysis of annotated isoforms models each transcript independently of the other transcripts coded by the gene. The relative analysis describes changes of specific regions or clusters associated with transcripts in the context of all transcripts that share that region. This consideration of intron clusters can account for unannotated transcript products that would have been ignored in the absolute analysis.

### 4.1. Absolute Differential Expression of Transcript Isoforms Associated with Opioid-Induced Hyperalgesia in the Nucleus Accumbens

The majority of the extreme differentially expressed transcript isoforms in the NAc were under-expressed in OIH relative to control mice (Table 1), whereas this pattern was reversed in the TG. The lower profiles associated with OIH could be related with NAc plasticity leading to morphine tolerance and hindrance of analgesic pathways that lower the transmission of pain signals. 

Transcript isoforms of Fxyd2 (ENSMUST000000213158.1), Pirt (ENSMUST000000123434.2), Acpp (ENSMUST00000062723.13), Avil (ENSMUST00000026500.11), Ntng1 (ENSMUST000000128219.8) were under-expressed (FDR-adjusted *P*-value < 5.0 × 10^–5^) in OIH relative to control mice (Table 1). The Fxyd2 pattern coincides with the down-regulation of Fxyd2 associated with the hyperexcitability of damaged sensory neurons in the dorsal root ganglia and neuropathic pain hypersensitivity in adult mice [47]. The Pirt profile at the isoform and gene levels [10] could be connected to the mechanical allodynia and thermal hyperalgesia reported in a Pirt knockout mouse line and conclusion that Pirt participates in neuropathic pain [48]. The observed Acpp isoform pattern is consistent with the gene-level profile [10] and is aligned with reports that injection of the ACPP protein inhibits nociception and with the suggestion that low levels of Acpp support prolonged pain sensation [49]. The pattern detected for the Avil matches reports that advillin knockout mice presented aggravated neuropathic pain [50]. The under-expression of Ntng1 in OIH mice could be associated with the low abundance of the NTNG1 protein in the cerebrospinal fluid of patients that experience chronic neuropathic pain that was not ameliorated by amitriptyline therapy [51]. 

Two transcript isoforms of Mpz (ENSMUST00000070758.9 and ENSMUST000000111334.1) were under-expressed in OIH relative to control mice (FDR-adjusted *P*-value < 2.9 × 10^–6^) and this profile was also reported in the gene level analysis [10]. The detected association between two transcript isoforms of Mpz and OIH could be linked to reports of more than 100 mutation in this gene that are associated with neuropathies and chronic pain [52]. 

Peripherin is a potentiator of NMDA-dependent calcium entry [53] and a transcript isoform of Prph (ENSMUST00000024249.4) was under-expressed in OIH relative to control mice. NMDA receptors participate in glutamatergic synapse and contribute to the induction and maintenance of central sensitization to stimuli and pain [54]. Also associated with sensitivity to stimuli and under-expressed in OIH at the isoform and gene levels [10] was Tusc5, a gene that is under-expressed after cold exposure and expressed in peripheral neurons [55]. At FDR-adjusted *P*-value < 5.0 × 10^–5^, Rgs7 was the only gene that had a transcript isoform over-expressed in OIH relative to control mice (Table 1). Congruent with the profile detected in the present study, a knockdown of Rgs7 strain presented enhanced potency of antinociception promoted by acute morphine administration [56].

### 4.2. Absolute Differential Expression of Transcript Isoforms Associated with Opioid-Induced Hyperalgesia in the Trigeminal Ganglia

The majority of the top differentially expressed transcript isoforms in the TG (Table 2) were over-expressed in OIH relative to control mice, a pattern opposite to NAc. This overarching profile suggests that hyperalgesia could be associated with heighten signaling that may support the transition from analgesia to hyperalgesia, and subsequent maintenance of the latter state. 

A transcript isoform of the A kinase (PRKA) anchor protein 11 gene (Akap11, ENSMUST000000227722.1, FDR-adjusted *P*-value < 7.8 × 10^–37^) was under-expressed in OIH relative to control mice. This pattern could be linked to the under-expression of Akap11 in human induced pluripotent sensory neurons exposed to Bortezomib, an anti-cancer medication that induces painful peripheral neuropathy [57]. The transcript isoform ENSMUST000000192394.5 of the gene muscleblind-like 1 (Mbnl1) was over-expressed in OIH relative to control mice in the TG (FDR-adjusted *P*-value < 6.2 × 10^–9^). The detected association between the Mbnl1 transcript isoform and OIH is complementary to the identification of Mbnl1 as a candidate gene for inflammatory nociception [58]. 

Many transcript isoforms that presented absolute differential expression between OIH and control mice in the TG, are alternative forms within the same gene (Table 2). Two transcript isoforms (ENSMUST000000118600.7 and ENSMUST000000118163.7) of Dmx-like2 (Dmxl2) were over-expressed in OIH relative to control mice (FDR-adjusted *P*-value < 1.2 × 10^–10^). The detected association between Dmxl2 and OIH could be associated with reports of up-regulation of the Dmxl2 protein in the spinal cord of a mouse model of neuropathic pain [59]. Likewise, multiple transcript isoforms within the Ral GTPase activating protein, alpha subunit 1 (Ralgapa1), pleckstrin and Sec7 domain containing 3 (Psd3), and microtubule-associated protein 2 (Map2) genes had significant associations with OIH. 

A transcript isoform (ENSMUST00000211368.1) belonging to phosphodiesterase 4 (Pde2a), an isoform ENSMUST00000196469.4 belonging to Nfkb1, and an isoform (ENSMUST00000151045.2) belonging to phosphodiesterase 4 (Gria1) were under-expressed in OIH relative to control mice in the TG (FDR-adjusted *P*-value < 0.07 and *P*-value < 0.01, respectively, Supplementary Table S2). This finding conforms with reports of the direct role of Pde4 family members in inflammatory processes involved in neuropathic pain [60]. The previous mode of action may be implicated in OIH because opioid exposure also disrupt inflammatory processes [60]. Likewise, the profile identified in this study could be associated with established participation of Nfkb1 in mediation of opioid analgesia [61]. 

The under-expression of a Gria1 transcript isoform could be aligned with the established role of Gria1 in neuropathic pain signaling among dorsal horn neurons, glutamate receptors signaling, and synaptic long-term potentiation [62]. Shank2 is member of the TG network (Figure 4) including an isoform (ENSMUST00000105900.8, FDR-adjusted *P*-value < 0.0007) that was over-expressed in OIH relative to control mice. The higher expression in OIH relative to control mice could be linked to reports of reduced acute nociception and chronic pain in a Shank2 knockout line relative to wild-type [63]. Moreover, Shank2 proteins play an important role in NMDA receptors-mediated pain hypersensitivity such that Shank2 mutations may suppress NMDA-ERK signaling in pain transmission [64], and this interaction has been liked to self-injurious behaviors in autism spectrum disorder. 

The Ophn1 transcript isoform ENSMUST00000147805 and the Ogt transcript isoform ENSMUST00000155792.1 were over-expressed in OIH relative to control mice (FDR-adjusted *P*-value < 0.006 and *P*-value < 0.0004, respectively, Supplementary Table S2) in the TG. The former finding could be associated with the effect of Ophn1 downregulation on the inhibition of glutamatergic synapse maturation, resulting in the loss of synaptic structure, function and plasticity which can lead to prolonged or enhanced sensitivity to pain [65]. The pattern of the latter transcript isoform could correspond with reports that Ogt knockout mic were hyposensitive to thermal and mechanical stimuli [66]. Three genes associated with rhythmic processes presented absolute differential isoform expression. While Dbp and Ciart were over-expressed at the isoform and gene levels [10], the transcript isoform from Arntl was under-expressed in OIH relative to control mice (Table 2), in agreement with the gene-level profile [10]. The role of alternative splicing on biological timing, and stress response has been reported in multiple species [67]. 

Transcript isoforms in the dynamin genes Dnm3 (ENSMUST00000193110.1), Dnm2 (ENSMUST00000169194.8), Dnm1 (ENSMUST00000230022.1) presented absolute differential expression between OIH and control mice in the TG. While the Dnm3 isoform was over-expressed (FDR-adjusted *P*-value < 0.05) in OIH, the isoforms of Dnm2 and Dnm1 were under-expressed (FDR-adjusted *P*-value < 0.08) in OIH relative to control mice. The dynamin gene family is known for experiencing extensive alternative splicing and participate in synaptic vesicle recycling, post-synaptic receptor internalization, neurosecretion and neuronal process extension, processes associated with stimuli transmission [68]. 

Notably, two transcript isoforms of Ral GTPase activating protein, alpha subunit 1 (Ralgapa1, isoforms ENSMUST00000220367.1 and ENSMUST00000226244.1) had opposite expression profiles between OIH and control mice (Table 2). Splicing variants of Ralgapa1 were detected in zebrafish, annotated to variants in human and mouse, and differentially associated with epilepsy [69]. 

### 4.3. Relative Differential Expression of Transcript Isoforms Associated with Opioid-Induced Hyperalgesia in the Nucleus Accumbens

The relative differential expression of St8sia1 isoforms associated with OIH identified in the NAc was characterized by the over-expression of a transcript isoform that just includes a 3′ exon in OIH relative to control mice (Table 3, Figure 1). The association between St8sia1 transcripts and OIH could be connected to the differential expression of St8sia in the dorsal root ganglion of a rat model of hyperalgesia elicited by repeated cold stress [70]. The protein coded by the St8sia1 gene has two transmembrane regions, one located near the 3′end, and transcript isoforms lacking this region may code for unstable peptides that may not be bioactive Also, the cryptic exon-skipping isoform of St8sia1, fragments the biological active domain of the Glycosyltransferase family 29 [71].

The alternative splicing of Cadps was characterized by the under-expression of an isoform that excludes an intermediate exon in OIH relative to control mice in the NAc (Table 3, Figure 1). Dysregulation of Cadps has been reported in the dorsal spinal cord of a rat model of chronic post-surgical pain [72]. The protein encoded by the Cadps exon-skipping isoform could have a compromised active domain that interferes with the Cadps participation in the synaptic vesicle exocytosis. 

The alternative splicing of Mief1 in the NAc associated with OIH was characterized by the under-expression of a transcript isoform that includes a lengthy 5′ exon in OIH relative to control mice (Table 3, Figure 1). Although there is no known direct connection between Mief1 and phenotypes akin to OIH, Mief1 participates in cell death [73]. The 5′ exon is important in the function of Mief1 because the transmembrane region of the coded protein is located near the 5′ end and disruption of this section may interfere with the function of ADP/GDP binding and the participation in the biological process of regulation of protein targeting to membrane and cellular response to hypoxia.

### 4.4. Relative Differential Expression of Transcript Isoforms Associated with Opioid-Induced Hyperalgesia in the Trigeminal Ganglia

Notable finding from the regional comparison of the differential alternative splicing between OIH and control mice was that TG presented more extreme splicing characterized by more extreme differences in the proportion of transcript isoforms between treatments relative to NAc (7 versus 2 genes at FDR-adjusted *P*-value < 0.05, Table 3). On the other hand, NAc presented more alternatively spliced genes at *P*-value < 0.0005 than TG (40 versus 32 genes, respectively). These differences in splicing profiles suggest that the role of alternative splicing underlying OIH is region-dependent.

Among the genes presenting relative differential isoform expression associated with OIH in the TG was Map2 (Table 4). The more extreme change in transcript isoform proportions encompassed under-expression in OIH relative to control mice. The detection of alternative splicing underlying the association of Map2 and OIH further advances the understanding of the role of this gene in pain. The under-expression in OIH detected in the present study could be linked to reports of decline in MAP-2 neurons in response to surgical injury that elicits pain behaviors [74,75,76].

The relative differential isoform expression of Fryl in the TG was characterized by the over-expression in OIH relative to control mice of a transcript that extends across two exons (Table 3, Figure 2), encompassing multiple MOR2-PAG1 domains across the sequence. Shorter transcript isoforms may exclude domains that are necessary for the bioactivity of the protein. Fryl has also been associated with functional genes clusters including zinc finger, DNA-binding, and regulation of transcription processes that are enriched in the present study [77]. Although associations between Fryl and noxious stimuli have not been established, a study of rats that self-administered cocaine identified detected over-expression of Fryl in the NAc [78].

The alternative splicing identified in the TG for Eml6 was characterized by the under-expression in OIH relative to control mice of a transcript isoform that extends across two exons (Figure 2, Table 3). The coded protein encompasses multiple WD40 repeats and belongs to the WD40 repeat EMAP family and the WD40 domain participates in pre-mRNA processing. The detected pattern suggests that OIH may enhance the coding of shorter forms of Eml6 that includes fewer WD repeats, potentially interfering with the molecular functions of the peptide in microtubule binding or mRNA preprocessing. 

The relative differential isoform expression identified in the TG for Ktn1, was characterized by the under-expression of a transcript that extends across two exons in OIH relative to control mice (Figure 2, Table 3). The association of Ktn1 with chronic pain has been proposed through the common association with putamen subcortical structure [79]. Ktn1 includes numerous coiled coil structures across the sequence and shorter transcript isoforms in the OIH group may code for peptides that have fewer coiled coils interfering with the molecular function of kinesin binding.

The significant alternative splicing identified in the TG for AW554918 was characterized by the under-expression of a transcript isoform that extends across two 3′ exons in OIH relative to control mice (Figure 2, Table 3). The excision of the AW554918 transcript results in nonsense mediated decay or processed transcript that alters the abundance and activity of the encoded protein [80]. A shortened AW554918 transcript isoform may affect the availability of the hinderin (Kiaa1328) protein to engage in the formation of multimeric protein complexes [80]. Cacna2d1 presented relative differential isoform expression in the TG and the change in the proportion of two transcripts between OIH and control mice presented opposite patterns (*P*-value < 0.003, Supplementary Table S3). The association of Cacna2d1 with OIH could correspond with reports of differential expression in mouse experiencing neuropathic pain [81].

### 4.5. Functional Analysis of Genes Presenting Alternative Splicing in Association with Opioid-Induced Hyperalgesia

The identification of enriched pathways encompassing genes that had absolute and relative differential isoform profiles offered insights into the alternative splicing action mode within and across the two brain regions studied (Table 4). A noteworthy result was the enrichment of the VEGF signaling pathway in the TG among genes presenting both absolute and relative differential isoform expression. This scenario indicates that differential alternative splicing could be required to change the level of the transcript isoform that supports differential expression between OIH and control mice. Our finding is aligned with reports that targeting VGF system of signaling and receptor molecules can ameliorate osteoarthritis pain [82], and is associated with neuropathic pain [83,84].

The assessment of enriched functional categories also uncovered pathways of genes presenting either one splicing mode of action in both regions such as PDGF signaling, integrin signaling, axon guidance, inflammation mediated by chemokine and cytokine signaling, p38 MAPK, Alzheimer disease, Ras signaling, and GABA signaling. The coordinated association between the previous pathways and OIH across regions magnifies the evidence that these molecular networks participate on hyperalgesia signaling. The identification of PDGF, integrin, and inflammation pathways is compatible with reports that PDGF activates nociceptive neurons and contributes to inflammatory pain [85], that PDGF signaling of receptors in the spinal immune cells elicits tactile allodynia [86], and the established role of integrin pathway in inflammatory and neuropathic pain [87]. 

Unique to the NAc, we detected absolute and relative differential splicing in the neuropeptide and neurotransmitter systems of gondadotropin-releasing, thyrotropin, oxytocin, and serotonin. Unique to the TG were absolute and relative isoform expression changes in circadian clock, insulin/glucose-related, and canabinoid signaling pathways (Table 4). Our findings are aligned with results from a study of a rat model of neuropathic pain that detected associations between axon guidance, circadian entrainment and insulin secretion and pain [88]. Similar to OIH, neuropathic pain presents heightened sensitivity of primary and secondary afferent neurons [5]. Our results also align with findings of the role of spinal cord neuropeptides such as thyrotropin releasing hormone and oxytocin in pain sensitivity and analgesia [89]. Going beyond previous reports of pathway enrichment, our findings offer a comprehensive understanding of the incidence of two alternative splicing action modes on the molecular mechanisms of pain.

### 4.6. Insights from the Integrated Study of Absolute and Relative Splicing Associated with Opioid-Induced Hyperalgesia

The simultaneous consideration of the genes that presented absolute or relative differential splicing across regions further the understanding of the disruption of splicing patterns associated with OIH (Table 5 and Table 6, Figure 3). Results from the analysis of relative differential isoform expression between OIH and control mice complemented the insights gained from the test for absolute differential expression. While the test on absolute levels identified changes in the individual transcript isoform between OIH and control mice, the test on relative levels identified proportional changes among the transcript isoforms in a cluster. Among the 43 genes that presented two alternative splicing events (the same splicing mechanisms in two regions or two mechanisms in the same region), five genes were annotated to the Gene Ontology Biological Process of RNA splicing and regulation of neurogenesis where enriched. The genes annotated to RNA splicing included Celf3, Rbm39, Hnrnpa1, Hnrnpk, and Prpf40b, and the genes associated with neurogenesis included Cdon, Grin1, and Hnrnpk. 

Our study of absolute and relative action modes further the understanding of the association between OIH and alternative splicing in Hnrnpk. Hnrnpk presented significant relative splicing in the NAc, and the most extreme DPSI was positive. This pattern indicates that a Hnrnpk transcript was relatively more abundant in the NAc of OIH relative to control mice (Supplementary Table S3). Also, a Hnrnpk transcript had absolute differential expression (FDR-adjusted *P*-value < 0.15) yet this transcript was under-expressed in the TG of OIH relative to control mice. The opposite profiles of absolute and relative Hnrnpk transcript isoform across brain regions are could be linked to reports of morphine-induced accumulation of HNRNPK protein in mouse brain regions correlated with nociceptive and antinociceptive modulatory systems [90].

One module of the NAc gene network (Figure 4A) encompasses the transcription modulator genes transcription factor 4 (Tcf4), transcription factor E2-alpha (Tcf3) and DNA excision repair protein ERCC-8 (Ercc8), interact with eukaryotic initiation factor 4A-II (Eif4a2). While Ercc8 is a component of the E3 ubiquitin-protein ligase complex that participates in transcription-coupled nucleotide excision repair [91], Eif4a2 plays a role in translation-related gene [92,93,94]. This network module is consistent with the enrichment of RNA splicing and RNA metabolism biological processes previously identified (Table 6) and further exposes the dysregulation of RNA splicing processes associated with OIH. In the remaining NAc network module (Figure 4A), encompass genes in the NMDA receptor system and other genes in this system are depicted in the TG network (Figure 4B). Grin encodes a NMDA glutamate receptor, and our finding could be connected with the established role of NMDA receptors in chronic pain and neural plasticity [95]. Also, Grin1 stabilizes and potentiates the signaling of the mu-opioid receptor that plays a critical role in the analgesia and subsequent hyperalgesia elicited by opioids [96]. This gene network is consistent with the enrichment of axon-, GABA- and neurogenesis functional categories previously identified (Table 4 and Table 6), highlighting the dysregulation of neural plasticity processes associated with OIH.

All the genes in the TG network were associated with the NMDA receptor system. In the sensory neurons, the NMDA receptor system plays a key role in the generation and maintenance of central sensitization during pain states [64]. Dysfunction of NMDA receptors in sensory neurons can cause deficits in pain responses, and NMDA receptors directly or indirectly bind to Shank proteins located at synapses in the CNS [64]. 

The identification of transcription factors pre B cell leukemia homeobox 1 (Pbx1) and aryl hydrocarbon receptor nuclear translocator like 2 (Arntl2), that could potentially target many genes presenting alternative splicing events enhanced the understanding of molecular mechanisms associated with OIH (Table 5). The role of these master regulators is aligned with reports Pbx1 was over-expressed in the striatum of rats that self-administered cocaine [97] and Arntl2 was under-expressed in a mouse knockout model of thermal hyperalgesia and mechanical allodynia [98]. 

The study of alternative splicing processes in a genome-wide scale using complementary approaches pointed to more than 150 isoforms and genes associated with OIH in two CNS regions. While the isoform findings were not experimentally validated, filtering criteria aimed at securing adequate isoform depth that would strengthen the findings. Also, the promising leads were discussed in the context of selective statistical and biological significance and supporting literature review in relevant pain and opioid-associated pathways while references at the gene level inidicate the need for further study at the isoform level across sexes, pre-existing pain conditions, and CNS regions.

## 5. Conclusions

The present study offers insights into the prevalent role of alternative splicing in the molecular mechanisms associated with OIH elicited by chronic morphine exposure. Both, changes in the absolute level of the transcript isoform and in the relative proportion of the transcript isoforms within a gene were detected in association with OIH in CNS regions that participate in stimuli signal transmission and opioid dependency. These findings can augment the accuracy in the identification of effect targets to reduce the effects of OIH.

## Figures and Tables

**Table 1:** Absolute differential expression of transcript isoforms (|fold change| > 2, FDR (false discovery rate)-adjusted *P*-value < 5.0 × 10^–5^ ) between opioid-induced hyperalgesia and control mice in the nucleus accumbens.

Gene Symbol	Gene Name	Transcript Identifier ^1^	LFC ^2^	FDR *P*-Value ^3^
Fxyd2	FXYD domain-containing ion transport reg. 2	213158.1	–2.08	2.0 × 10^–15^
Tusc5	tumor suppressor candidate 5	62024.2	–1.40	2.3 × 10^–14^
Tmem233	transmembrane protein 233	111997.1	–1.47	1.7 × 10^–12^
Zbtb7b	zinc finger and BTB domain containing 7B	107433.7	–8.55	1.2 × 10^–11^
Pirt	phosphoinositide–interacting regulator of trans.	123434.2	–1.29	1.8 × 10^–9^
Sncg	synuclein, gamma	23826.4	–1.27	1.5 × 10^–8^
Mpz	myelin protein zero	70758.9	–1.30	1.5 × 10^–8^
Acpp	acid phosphatase, prostate	62723.13	–1.64	2.5 × 10^–8^
Avil	advillin	26500.11	–1.15	4.1 × 10^–8^
Rab2b	RAB2B, member RAS oncogene family	100631.1	–9.26	5.7 × 10^–8^
Tspan8	tetraspanin 8	80630.1	–3.74	9.3 × 10^–8^
Ntng1	netrin G1	128219.8	–0.62	6.4 × 10^–7^
Cdh1	cadherin 1	312.11	–1.08	9.0 × 10^–7^
Pmp22	peripheral myelin protein 22	108702.7	–1.41	1.1 × 10^–6^
Mpz	myelin protein zero	111334.1	–1.24	2.9 × 10^–6^
Hnrnpa1	heterogeneous nuclear ribonucleoprotein A1	36004.15	–1.82	3.2 × 10^–6^
Prr13	proline rich 13(Prr13)	164688.1	–7.54	1.3 × 10^–5^
Prrxl1	paired related homeobox protein–like 1	189022.7	–1.29	1.3 × 10^–5^
Dysf	dysferlin	204591.2	–8.72	1.3 × 10^–5^
Prph	peripherin	24249.4	–1.11	1.3 × 10^–5^
Rab3d	RAB3D, member RAS oncogene family	122211.7	–6.99	1.3 × 10^–5^
Brap	BRCA1 associated protein	111765.7	–6.95	1.7 × 10^–5^
Rgs7	regulator of G protein signaling 7	27812.1	8.41	1.9 × 10^–5^
Ppp1r1c	protein phosphatase 1, regulatory subunit 1C	111780.2	–2.83	1.9 × 10^–5^
Shroom3	shroom family member 3	113055.8	–6.96	2.6 × 10^–5^

^1^ Abbreviated transcript identifier = ENSMUST000000 (ENS, species and object type) precedes the complete Ensembl identifier. ^2^ LFC = log2(fold change between opioid-induced hyperalgesia and control mice). ^3^ FDR-adjusted *P*-value.

**Table 2:** Absolute differential expression of transcript isoforms (|fold change| > 2, FDR-adjusted *P*-value < 5.0 × 10^–5^) between opioid-induced hyperalgesia and control mice in the trigeminal ganglia.

Gene Symbol	Gene Name	Transcr. Identity ^1^	LFC ^2^	FDR *P*-Value ^3^
Akap11	A kinase (PRKA) anchor protein 11	227722.1	–9.04	7.8 × 10^–37^
Dbp	D site albumin promoter binding protein	80885.11	1.23	5.8 × 10^–21^
Zfp871	zinc finger protein 871	159086.8	1.48	9.3 × 10^–19^
Dmxl2	Dmx-like 2	118600.7	1.29	1.0 × 10^–18^
RP24-200D3.4	predicted gene, 38394	179598.3	1.47	1.2 × 10^–10^
Dmxl2	Dmx-like 2	118163.7	2.29	1.2 × 10^–10^
Bdp1	B double prime 1, transcription initiation fact.	38104.11	1.43	2.5 × 10^–10^
Ralgapa1	Ral GTPase activating protein, alpha subunit 1	220367.1	–0.89	6.9 × 10^–10^
Ciart	circadian associated repressor of transcription	36418.9	1.28	1.8 × 10^–9^
RP23-423B1.6	predicted gene, 38020	193066.1	1.43	1.8 × 10^–9^
RP23-243E17.1	RIKEN cDNA 4932438A13	152564.7	1.25	6.2 × 10^–9^
Mbnl1	muscleblind-like 1	192394.5	1.19	6.2 × 10^–9^
Atp13a5	ATPase type 13A5	75806.1	1.04	1.1 × 10^–7^
Arntl	aryl hydrocarbon receptor nuclear translo.-like	47321.8	–1.15	1.7 × 10^–7^
Ralgapa1	Ral GTPase activating protein, alpha subunit 1	226244.1	1.07	2.8 × 10^–7^
Map2	microtubule-associated protein 2	114013.7	1.06	3.3 × 10^–7^
Grip1	glutamate receptor interacting protein 1	105261.8	4.95	7.2 × 10^–7^
Tert	telomerase reverse transcriptase	22104.8	3.28	9.0 × 10^–7^
Lcor	ligand dependent nuclear receptor corepressor	67795.11	1.23	1.1 × 10^–6^
Psd3	pleckstrin and Sec7 domain containing 3	98696.9	1.72	1.6 × 10^–6^
Rfx4	regulatory factor X, 4	166696.8	–5.58	2.7 × 10^–6^
Fabp7	fatty acid binding protein 7, brain	165013.1	–1.00	3.0 × 10^–6^
Scn9a	sodium channel, voltage-gated, type IX, alpha	169900.7	1.68	3.0 × 10^–6^
Psd3	pleckstrin and Sec7 domain containing 3	93468.11	2.48	8.2 × 10^–6^
Tia1	cytotoxic granule-associat. RNA bind. prot. 1	136387.1	1.00	1.2 × 10^–5^
Map2	microtubule-associated protein 2	114018.9	1.01	1.5 × 10^–5^
Wnk3	WNK lysine deficient protein kinase 3	184730.7	2.03	1.6 × 10^–5^
Snrpa	small nuclear ribonucleoprotein polypep. A	163311.8	7.63	2.9 × 10^–5^

^1^ Abbreviated transcript identifier = ENSMUST000000 (ENS, species and object type) precedes the complete Ensembl identifier. ^2^ LFC = log_2_(fold change between opioid-induced hyperalgesia and control mice). ^3^ FDR-adjusted *P*-value.

**Table 3:** Relative differential expression of transcript isoform clusters (FDR-adjusted *P*-value < 0.1) between opioid-induced hyperalgesia and control mice in the nucleus accumbens (NAc) and trigeminal ganglia (TG).

Region Gene Symbol	Gene Name	ΔPSI Range ^1^		Count ^2^	FDR *P*-Value
*NAc*					
Cadps	Ca^2+^-dependent secretion activator	–0.080	0.108	6	3.2 × 10^–2^
Rab28	RAB28, member RAS oncogene family	–0.060	0.067	4	3.2 × 10^–2^
Trak2	trafficking protein, kinesin binding 2	–0.026	0.013	4	8.0 × 10^–2^
Grin1	glutamate receptor, ionotropic, NMDA1	–0.011	0.010	6	9.9 × 10^–2^
Pex5l	peroxisomal biogenesis factor 5-like	–0.040	0.044	14	9.9 × 10^–2^
Gm3764	predicted gene 3764	–0.099	0.066	7	9.9 × 10^–2^
St8sia1	ST8 sialyltransferase 1	–0.097	0.119	3	9.9 × 10^–2^
Xiap	X-linked inhibitor of apoptosis	–0.081	0.052	6	9.9 × 10^–2^
Uxs1	UDP-glucuronate decarboxylase 1	–0.034	0.051	3	9.9 × 10^–2^
Mbp	myelin basic protein	–0.012	0.023	3	9.9 × 10^–2^
Mief1	mitochondrial elongation factor 1	–0.127	0.116	3	9.9 × 10^–2^
*TG*					
Fryl	FRY like transcription coactivator	–0.331	0.634	3	1.1 × 10^–4^
S100a6	S100 calcium binding protein A6	–0.004	0.007	5	8.4 × 10^–4^
Map2	microtubule-associated protein 2	–0.088	0.065	13	8.4 × 10^–4^
Eml6	echinoderm microtubule protein like 6	–0.663	0.231	5	2.4 × 10^–3^
Ktn1	kinectin 1	–0.100	0.059	3	3.7 × 10^–3^
AW554918	expressed sequence (hinderin)	–0.148	0.103	5	4.1 × 10^–2^
Clasp2	CLIP associating protein 2	–0.025	0.033	6	4.3 × 10^–2^
Sdccag8	serologic. defined colon cancer antigen 8	–0.054	0.101	6	7.7 × 10^–2^

^1^ΔPSI range = the most extreme positive and negative ΔPSI among all the transcript isoforms in the gene cluster are reported. A positive ΔPSI identifies a higher transcript isoform proportion in OIH relative to control mice while a negative ΔPSI identifies a lower transcript isoform proportion in OIH relative to control mice. Count^2^ = number of transcript isoforms in the intron clusters of the gene.

**Table 4:** Enriched PANTHER pathways among genes presenting absolute or relative differential isoform expression (*P*-value < 0.001, FDR-adjusted *P*-value < 0.1) between opioid-induced hyperalgesia (OIH) and control (CON) mice in the nucleus accumbens (NAc) and trigeminal ganglia (TG) using the Over-Representation Analysis (ORA) and Gene Set Enrichment Analysis (GSEA) approaches.

Region, Type, Analysis ^1^	Path ^2^	Pathway Name	Size ^3^	Enr. Score ^4^	*P*-Value
*NAc absolute differential isoform expression*					
ORA	P00047	PDGF signaling pathway	115	2.39	5.1 × 10^–3^
ORA	P00049	Parkinson disease	85	2.65	5.8 × 10^–3^
ORA	P00034	Integrin signalling pathway	154	2.11	6.9 × 10^–3^
*NAc relative differential isoform expression*					
ORA	P05918	p38 MAPK pathway	35	9.59	3.3 × 10^–3^
ORA	P00043	Muscarinic acetylcholine recep. 2–4 signal.	50	6.71	9.1 × 10^–3^
GSEA	P06664	Gonadotropin-releasing hormone receptor	119	–2.31	0.0E+00
*TG absolute differential isoform expression*					
ORA	P00015	Circadian clock system	9	13.73	1.1 × 10^–5^
ORA	P00047	PDGF signaling pathway	115	2.58	1.8 × 10^–3^
ORA	P00009	Axon guidance mediated by netrin	28	4.41	4.5 × 10^–3^
ORA	P00008	Axon guidance mediated by Slit/Robo	18	5.49	4.9 × 10^–3^
ORA	P00034	Integrin signalling pathway	154	2.09	7.6 × 10^–3^
ORA	P00003	Alzheimer disease-amyloid secretase	60	2.88	9.4 × 10^–3^
*TG relative differential isoform expression*					
GSEA	P00056	VEGF signaling pathway	40	2.04	3.1 × 10^–3^
GSEA	P05730	Endogenous cannabinoid signaling	12	−1.96	4.1 × 10^–3^
GSEA	P00041	Metabotropic glutamate receptor group I	13	−1.85	7.1 × 10^–3^

^1^ Region = NAc or TG; Type = absolute or relative differential isoform expression; Analysis = ORA or GSEA. ^2^ Path = pathway identifier. ^3^ Size = number of distinct genes in the pathway. ^4^ Enr score = enrichment score (ORA observed/expected ratio or GSEA Normalized Enrichment Score); NES > 0 (or NES < 0) most extreme isoform proportion over-expressed (or under-expressed) in OIH relative to control mice.

**Table 5:** Genes presenting simultaneously two splicing modes of action (absolute-DE, or relative-AS) in one region (nucleus accumbens-NAc, or trigeminal ganglia-TG) or one mode of action in two regions.

Group ^1^	Genes Detected in Two Differential Splicing-Region Categories ^2^
NAc	Prpf40b,Grin1,Ablim2,Rbm3,Hebp2,Ciz1,Dhx30,Fam219a,Hnrnpa1
TG	Asap1,Scn8a,Eef1d,Map2,Rbm39,Cnot1,Fabp7,Ciart,Fam222b,Peg3
DE	Slc38a4,Pak3,Prg4,Csde1,Celf3,Cacna1g,Ndst3,AC166328.1,Mapk8ip3
AS	4933431E20Rik,Rasl10a,Fabp7,Ssbp2,Hnrnpk,Plce1,Tenm4,Cdon,Zfp948, Rbm3,Clasp2,Spag9,Ptpn23,Mprip,Cadps,Mink1,Prune2,Asap1,Fam184a, Podxl2,Sema4c,Smpdl3a

^1^ Group = genes presenting simultaneous absolute and relative splicing action modes in NAc or TG, and genes presenting either DE or AS action simultaneously in both regions. ^2^ Gene names: 4933431E20Rik = RIKEN cDNA 4933431E20 gene; Ablim2 = actin-binding LIM protein 2; Asap1 = ArfGAP with SH3 domain, ankyrin repeat and PH domain1; Cacna1g = calcium channel, voltage-dependent, T type, alpha 1G subunit; Cadps = Ca2+-dependent secretion activator; Cdon = cell adhesion molecule-related/down-regulated by oncogenes; Celf3 = CUGBP, Elav-like family member 3; Ciart = circadian associated repressor of transcription; Ciz1 = CDKN1A interacting zinc finger protein 1; Clasp2 = CLIP associating protein 2; Cnot1 = CCR4-NOT transcription complex, subunit 1; Csde1 = cold shock domain containing E1, RNA binding; Dhx30 = DEAH (Asp-Glu-Ala-His) box polypeptide 30; Eef1d = eukaryotic translation elongation factor 1 delta; Fabp7 = fatty acid binding protein 7, brain; Fam184a = family with sequence similarity 184, member A(Fam184a); Fam219a = family with sequence similarity 219, member A; Fam222b = family with sequence similarity 222, member B; Grin1 = glutamate receptor, ionotropic, NMDA1 (zeta 1); Hebp2 = heme binding protein 2; Hnrnpa1 = heterogeneous nuclear ribonucleoprotein A1(; Hnrnpk = heterogeneous nuclear ribonucleoprotein K; Map2 = microtubule-associated protein 2; Mapk8ip3 = mitogen-activated protein kinase 8 interacting protein 3; Mink1 = misshapen-like kinase 1; Mprip = myosin phosphatase Rho interacting protein; Ndst3 = N-deacetylase/N-sulfotransferase 3; Pak3 = p21 protein (Cdc42/Rac)-activated kinase 3; Peg3 = paternally expressed 3; Plce1 = phospholipase C, epsilon 1; Podxl2 = podocalyxin-like 2; Prg4 = proteoglycan 4; Prpf40b = pre-mRNA processing factor 40B(Prpf40b); Prune2 = prune homolog 2; Ptpn23 = protein tyrosine phosphatase, non-receptor type 23; Rasl10a = RAS-like, family 10, member A Rbm3 = RNA binding motif protein 3; Rbm39 = RNA binding motif protein 39; Scn8a = sodium channel, voltage-gated, type VIII, alpha; Sema4c = sema domain, immunoglobulin domain (Ig), transmembrane domain (TM) and short cytoplasmic domain, (semaphorin) 4C; Slc38a4 = solute carrier family 38, member 4; Smpdl3a = sphingomyelin phosphodiesterase, acid-like 3A; Spag9 = sperm associated antigen 9; Ssbp2 = single-stranded DNA binding protein 2; Tenm4 = teneurin transmembrane protein 4; Zfp948 = zinc finger protein 948;

**Table 6:** Enriched Gene Ontology biological process category among the genes presenting absolute and relative differential isoform expression in the nucleus accumbens and trigeminal ganglia identified using Over-Representation Analysis.

Category	Category Name	Count ^1^	Enrich. Score ^2^	*P*-Value	FDR *P*-Value
GO:0050767	regulation of neurogenesis	11	5.99	1.2 × 10^–6^	1.1 × 10^–2^
GO:0016071	mRNA metabolic process	9	7.29	2.8 × 10^–6^	1.2 × 10^–2^
GO:0008380	RNA splicing	6	8.35	7.4 × 10^–5^	6.3 × 10^–2^
GO:0043410	positive regulation of MAPK cascade	7	6.46	8.8 × 10^–5^	6.3 × 10^–2^

^1^ Count = number of distinct genes in the category. ^2^ Enrichment score.

**Table 7:** Enriched transcription factors among target genes presenting absolute or relative splicing action modes between opioid-induced hyperalgesia and control mice in the trigeminal ganglia.

Transcription Factor	NES ^1^	Target Gene Count ^2^	Motif ^3^	*P*-Value
Pbx1	4.00	31	2	1.0 × 10^–3^
Arntl2	3.73	55	4	3.8 × 10^–3^

^1^ NES = normalized enrichment score. ^2^ Target Gene Count = number of target genes presenting absolute or relative differential isoform expression. ^3^ Motif = number of motifs in the transcription factor that correspond to regulatory regions in the genes.

## Data Availability

Data is available from the Gene Expression Omnibus (GEO) database (experiment identifier GSE126662).

## Supplementary Materials

The following are available online at https://www.mdpi.com/article/10.3390/genes12101570/s1, Table S1: Absolute differential isoform expression (*P*-value < 5.0 × 10^–2^) between opioid-induced hyperalgesia and control mice in the nucleus accumbens., Table S2: Absolute differential isoform expression (*P*-value < 5.0 × 10^–2^) between opioid-induced hyperalgesia and control mice in the trigeminal ganglia., Table S3: Relative differential isoform expression (*P*-value < 0.0005) between opioid-induced hyperalgesia relative and control mice in the nucleus accumbens (NAc) and trigeminal ganglia (TG)., Table S4: Enriched PANTHER database pathways among isoforms presenting differential (*P*-value < 0.005) expression (DE) or splicing (DS) between opioid-induced hyperalgesia (OIH) and control (CON) mice in the nucleus accumbens (NAc) and trigeminal ganglia (TG) using Over-Representation Analysis (ORA) and Gene Set Enrichment Analysis (GSEA) approaches.
